# Serum hepatitis B virus large and medium surface proteins as novel tools for predicting HBsAg clearance

**DOI:** 10.3389/fimmu.2022.1028921

**Published:** 2022-09-23

**Authors:** Xiao Lin, Yanhong Zheng, Hong Li, Junfeng Lu, Shan Ren, Yisi Liu, Xiaoxiao Wang, Sujun Zheng, Lina Ma, Zhenhuan Cao, Xinyue Chen

**Affiliations:** ^1^ First Department of Liver Disease Center, Beijing Youan Hospital, Capital Medical University, Beijing, China; ^2^ Third Department of Liver Disease Center, Beijing Youan Hospital, Capital Medical University, Beijing, China

**Keywords:** inactive HBsAg carriers, pegylated interferon, HBsAg clearance, LHBs, MHBs, predictor

## Abstract

**Background:**

There is still lack of reliable predictors for hepatitis B surface antigen (HBsAg) clearance. Recent studies have shown that the levels of large (LHBs) and medium hepatitis B surface proteins (MHBs) are closely related to antiviral efficacy. This study aimed to investigate the possibility of LHB and MHB levels to predict HBsAg clearance.

**Methods:**

An inactive HBsAg carriers (IHCs) cohort that had received pegylated interferon (Peg-IFN) treatment was divided into the HBsAg-cleared group (R group) and the HBsAg non-cleared group (NR group) based on whether HBsAg was cleared at 96 weeks. We detected the levels of LHBs and MHBs to evaluate the possibility of predicting HBsAg clearance.

**Results:**

There were 39 patients in the R group and 21 in the NR group. The total HBsAg, LHB, and MHB levels at baseline and at 12 weeks were significantly lower in the R group than in the NR group (all p< 0.05). Multivariate logistic regression indicated that LHB and MHB levels at baseline and 12 weeks were independent predictors of HBsAg clearance (OR = 0.435, p = 0.016; OR = 0.136, p = 0.003; OR = 0.137, p = 0.033; OR = 0.049, p = 0.043). The area under the curve (AUC) for the baseline and 12-week LHB and MHB levels was 0.827-0.896, which were greater than that of the total HBsAg level at baseline and 12-week (AUC: 0.654-0.755). Compared with the prediction results of a single indicator, the combination of LHB and MHB levels had better value in predicting HBsAg clearance. The AUCs of combination factor 1, constructed from baseline LHB and MHB, and combination factor 2, constructed from 12-week LHB and MHB, were 0.922 and 0.939, respectively, and the sensitivity (82.05%-100.00%) and specificity (85.71%-100.00%) were both high. The combined indicators based on baseline LHBs ≤ 13.99 ng/mL and MHBs ≤ 7.95 ng/mL predicted HBsAg clearance rate of more than 90%.

**Conclusion:**

Baseline and 12-week LHB and MHB levels can predict HBsAg clearance obtained by Peg-IFN therapy in IHCs, and the predictive value is higher than that of the total HBsAg levels.

## Introduction

Chronic hepatitis B virus (HBV) infection is an important global public health problem, especially in the Asia-Pacific region. In recent years, domestic and international chronic Hepatitis B (CHB) management guidelines have made hepatitis B surface antigen (HBsAg) clearance as the highest clinical treatment goal (1–3). Because of limited therapeutic drugs, improving prediction efficacy and thus providing precise treatment are key issues for improving the clinical cure rate. But the prediction of clinical cure with antiviral therapy for CHB has not been well established. HBsAg consists of large (LHBs), medium (MHBs) and small surface proteins, and their relative proportions are closely related to disease stage. Recent studies have reported that LHBs and MHBs are associated with HBsAg clearance and that the value of baseline MHBs in predicting clinical cure is better than that of total HBsAg (4). However, that was a small-sample study based on most patients received nucleos(t)ide analogue (NAs) therapy. We accumulated a large number of patients who achieved HBsAg clearance during treatment with pegylated interferon (Peg-IFN). To this end, we investigated the value of LHB and MHB levels in predicting HBsAg clearance in inactive HBsAg carriers (IHCs) treated with Peg-IFN.

## Materials and methods

### Study population

IHCs who had been treated with Peg-IFN in our hospital were included in this study, and blood samples were collected at baseline and at 12 weeks of treatment. All IHCs met the criteria defined in the prevention and treatment guidelines for chronic hepatitis B (2019 edition) (3). HBsAg positive > 6 months and HBsAg<1000 IU/mL, HBeAg negative, anti-HBe positive; HBV DNA<2000 IU/mL, and ALT and AST remained normal; no signs of liver cirrhosis on imageology examination. Enrolled IHCs were treated with Peg-IFN 135 mg weekly by subcutaneous injection in our hospital, and the total duration of treatment was 96 weeks. Based on the results at 96 weeks of treatment, the patients were divided into the HBsAg-cleared group (responders group, R group) and the HBsAg non-cleared group (non-responders, NR group). Patients in the R group satisfied the following criteria: a sustained virological response (HBV DNA undetectable), hepatitis B e antigen (HBeAg) negative status, HBsAg clearance (HBsAg< 0.05 IU/ml) or seroconversion (hepatitis B surface antibody≥ 10 IU/ml). Those who did not meet any of the above indicators were included in the NR group. All enrolled patients signed an informed consent form, and the study was approved by the Ethics Committee of Beijing Youan Hospital affiliated with Capital Medical University ([2017]24).

### Detection indicators and methods

The levels of LHBs and MHBs were detected by enzyme-linked immunosorbent assay (Shanghai Jianglai Biotechnology Co., Ltd., China), the lower limit of detection was 0.5 ng/ml. HBV markers were detected using Elecsys MODULAR ANALYTICS E170 (Roche Diagnostics GmbH, Germany), the lower limit for the quantitative detection of HBsAg was <0.05 IU/ml, anti-HBs >10 IU/ml, and HBeAg >1 COI were considered positive. HBV DNA was detected using the cobas^®^ AmpliPrep/cobas^®^ TaqMan automatic nucleic acid isolation and purification system and PCR analysis system (Roche Diagnostics GmbH, Germany) with a lower limit of detection of 20 IU/ml. Alanine aminotransferase (ALT) was detected using reagents from Shanghai Kehua Dongling Company (China), and a normal value was considered< 40 U/L.

### Statistical analysis

SPSS 21.0 (SPSS, USA) and Medcalc Statistical Software version 14.8.1 (MedCalc Software Ltd, Ostend, Belgium) were used for statistical processing. The continuous variables were expressed as the mean ± standard deviation or median (interquartile range), and categorical variable data were expressed as the number of cases and the percentage [cases (%)]. Continuous parameters were analyzed by Student’s t-test or Mann-Whitney U test, categorical parameters were analyzed by the Pearson Chi-square test or Fisher’s exact test. Logistic regression analysis was conducted to identify factors associated with HBsAg clearance. Receiver Operating Characteristic (ROC) analysis was performed to analyze the predictive value of the factors in predicting HBsAg clearance. The cut-off value corresponding to the maximum Youden index is regarded as the best cut-off (Youden index = sensitivity + specificity - 1). P-value< 0.05 was considered significant.

## Results

### Patient characteristics

Sixty patients met the above criteria (39 patients in the R group and 21 patients in the NR group), all of whom were Asian. The average age (years) of patients in the R group was 38.05 ± 11.25 years and that of patients in the NR group was 40.62 ± 11.26 years. The proportions of males in the R group and the NR group were 69.23% (27/39) and 76.19% (16/21), respectively. There was no significant difference in age or sex between the two groups (p = 0.368, p = 0.568, **Table 1**).

**Table 1:** Characteristics of R and NR groups at baseline and at 12 weeks of treatment.

CParameter	All patients (n = 60)	R group (n = 39)	NR group (n = 21)	P-value
Gender (M/F)	43/17	27/12	16/5	0.568
Age (years)	38.95 ± 11.23	38.05 ± 11.25	40.62 ± 11.26	0.368
Baseline								
Total HBsAg, IU/ml	198.78 ± 236.54	155.63 ± 194.33	287.35 ± 291.79	0.046
LHBs, ng/ml	13.28 ± 1.89	12.49 ± 1.53	14.76 ± 1.59	<0.001
MHBs, ng/ml	7.19 ± 1.19	6.62 ± 0.88	8.23 ± 0.99	<0.001
ALT, U/L	31.31 ± 14.51	30.88 ± 14.62	33.17 ± 14.36	0.570
12 weeks								
Total HBsAg, IU/ml	91.93 ± 170.75	37.77 ± 57.15	189.92 ± 250.70	0.001
LHBs, ng/ml	11.23 ± 2.10	10.22 ± 1.64	13.09 ± 1.52	<0.001
MHBs, ng/ml	6.20 ± 1.14	5.70 ± 0.90	7.12 ± 0.94	<0.001
ALT, U/L	76.78 ± 77.74	82.56 ± 89.52	66.05 ± 49.14	0.681
ALT>1.0×ULN	53.33% (32/60)	56.41%(22/39)	47.62%(10/21)	0.515
ALT>1.5×ULN	41.67% (25/60)	51.28% (20/39)	23.81% (5/21)	0.040
ALT>2.0×ULN	23.33%(14/60)	28.20%(11/39)	14.29%(3/21)	0.224
Change from baseline to 12 weeks								
Total HBsAg, log IU/ml	-0.73 ± 0.87	-0.91 ± 0.96	-0.38 ± 0.50	0.021
LHBs, log ng/ml	-0.08 ± 0.06	-0.09 ± 0.07	-0.05 ± 0.04	0.015
MHBs, log ng/ml	-0.07± 0.06	-0.07 ± 0.07	-0.06 ± 0.06	0.821

HBsAg, Hepatitis B surface antigen; LHBs, large hepatitis B surface proteins; MHBs, middle hepatitis B surface proteins; ALT, alanine aminotransferase; ULN, upper limit of normal.

### Total HBsAg, LHB and MHB levels in the R group and the NR group

**Figure 1:**
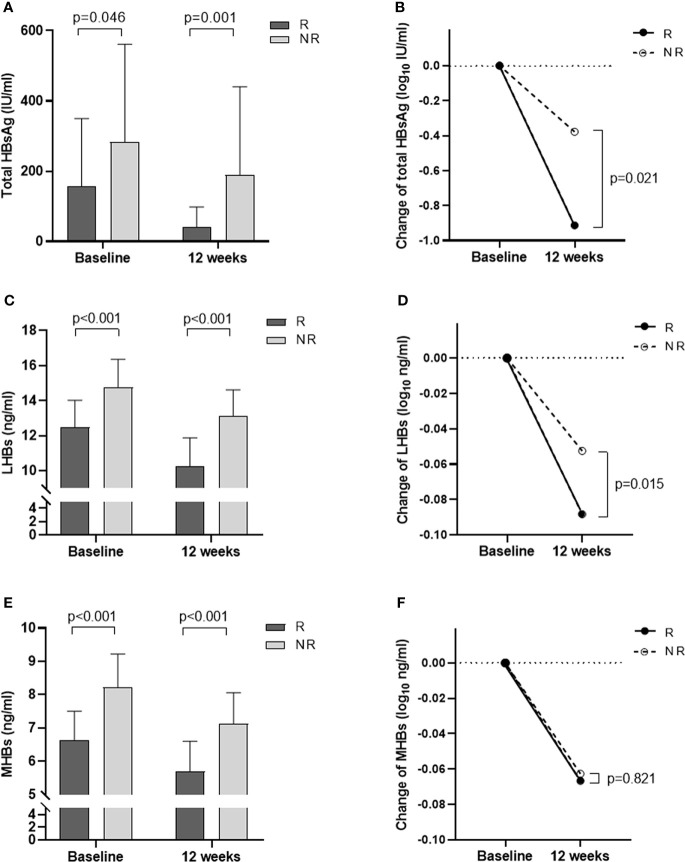
Changes in total HBsAg, LHBs, and MHBs at baseline and 12 weeks of treatment in the R and NR groups. **(A)** Total HBsAg levels at baseline and 12 weeks. **(B)** The degree of decline in total HBsAg from baseline to 12 weeks (log). **(C)** LHBs levels at baseline and 12 weeks. **(D)** The degree of decline in LHBs from baseline to 12 weeks (log). **(E)** MHBs levels at baseline and 12 weeks. **(F)** The degree of decline in MHBs from baseline to 12 weeks (log).

We measured the total HBsAg levels in the R group and the NR group at baseline and at 12 weeks. The results showed that the total HBsAg level in the R group was significantly lower than that in the NR group (all p< 0.05, **Figure 1A**, **Table 1**). At 12 weeks of Peg-IFN treatment, the total HBsAg level in the two groups decreased compared with the baseline, and the reduction in the R group was greater than that in the NR group (p = 0.021; **Figure 1B**).

Further analysis of LHB and MHB levels at baseline and 12 weeks showed that LHB and MHB levels in the R group were significantly lower than those in the NR group (all p< 0.001, **Figures 1C, E**, **Table 1**). At 12 weeks of treatment, the levels of LHBs and MHBs decreased compared to those at baseline. The reduction in LHBs was greater in the R group than in the NR group (p = 0.015, **Figure 1D**), while the reduction in MHBs was similar in the two groups (p = 0.821; **Figure 1F**).

### Analysis of related influencing factors of HBsAg clearance

#### Logistic regression

Logistic regression analysis was performed on the factors that may affect HBsAg clearance. Univariate logistic regression analysis showed that baseline LHB and MHB levels, 12-week total HBsAg, LHB and MHB levels, 12-week total HBsAg and LHB reduction (log), and 12-week ALT greater than 1.5 times the upper limit of normal (ULN) were all influencing factors of HBsAg clearance (**Table 2**). Multivariate logistic regression analysis showed that only baseline and 12-week LHB and MHB levels were influencing factors of HBsAg clearance [odds ratio (OR) = 0.435, p = 0.016; OR = 0.136, p = 0.003; OR = 0.137, p = 0.033; OR = 0.049, p = 0.043; **Table 2**].

**Table 2:** Logistic regression.

Parameter	Univariate logistic regression			Multivariate logistic regression		
	OR	95% CI	P-value	OR	95% CI	P-value
Sex	0.703	0.209 - 2.364	0.569	0.316	0.026 - 3.783	0.363
Age	0.980	0.934 - 1.027	0.397	0.933	0.840 -1.035	0.189
LHBs at baseline	0.364	0.211 - 0.627	<0.001	0.435	0.221 - 0.854	0.016
MHBs at baseline	0.157	0.063 - 0.392	<0.001	0.136	0.037 - 0.502	0.003
Total HBsAg at baseline	0.998	0.995 - 1.000	0.051	0.994	0.998 -1.000	0.070
LHBs at 12 weeks	0.375	0.231 - 0.610	<0.001	0.137	0.022-0.853	0.033
MHBs at 12 weeks	0.185	0.075 - 0.458	<0.001	0.049	0.003 -0.907	0.043
Total HBsAg at 12 weeks	0.991	0.985 - 0.998	0.007	0.989	0.975 - 1.003	0.128
LHBs decline at 12 weeks (log)	1.010	1.000-1.019	0.044	0.968	0.932-1.005	0.251
MHBs decline at 12 weeks (log)	1.001	0.993-1.009	0.817	0.980	0.948-1.014	0.241
Total HBsAg decline at 12 weeks (log)	1.001	1.000-1.002	0.030	1.002	0.999-1.006	0.431
ALT>1.5×ULN at 12 weeks	0.297	0.091 - 0.970	0.044	0.283	0.012 -6.580	0.033

1,R group; 0,NR group; 1,male; 0f,emale; 1,ALT>1.5×ULN at 12 weeks; 0,ALT ≤ 1.5×ULN at 12 weeks); HBsAg, Hepatitis B surface antigen; LHBs, large hepatitis B surface proteins; MHBs, middle hepatitis B surface proteins; ALT, alanine aminotransferase; ULN, upper limit of normal; OR, odds ratio; CI, confidence interval.

#### The predictive value of relevant influencing factors for HBsAg clearance by ROC evaluation

**Figure 2:**
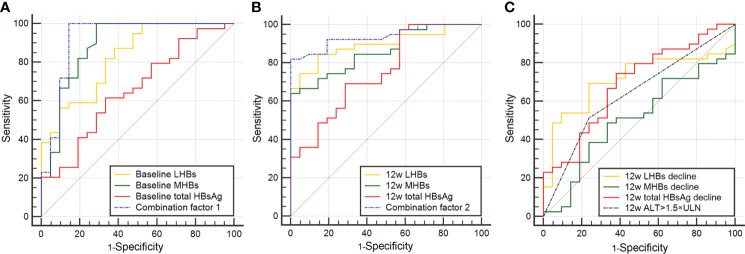
ROC curves of HBsAg clearance predictors. **(A)** ROC curves of total HBsAg, LHBs, MHBs, and combination factor 1 at baseline. **(B)** ROC curves of total HBsAg, LHBs, MHBs, and combination factor 2 at 12 weeks. **(C)** ROC curves of the degree of decline in total HBsAg, LHBs, and MHBs from baseline to 12 weeks, and ALT elevations >1.5 × ULN at 12 weeks.

Based on the logistic regression results, the ROC curve and area under the curve (AUC) for total HBsAg, LHB and MHB levels at baseline and 12 weeks, reductions (log), and ALT > 1.5 × ULN were calculated (**Table 3**). The AUCs of LHBs and MHBs at baseline were 0.827 and 0.887, respectively, which were greater than the AUC of the total HBsAg level (0.654, **Figure 2A**). The AUC for baseline MHBs was significantly higher than that for total HBsAg (p = 0.014, 95% CI: 0.048-0.417; **Table 3**), suggesting that the value of baseline MHBs in predicting HBsAg clearance was significantly better than that of total HBsAg. At 12 weeks of treatment, the AUCs for LHBs and MHBs were 0.896 and 0.863, respectively, which were higher than the AUC for the total HBsAg level (0.755, **Figure 2B**), but the difference was not statistically significant (p=0.082, p=0.179, **Table 3**). In addition, the AUC for the reduction in LHBs at 12 weeks was 0.711, which was higher than the AUCs for the reduction in the total HBsAg and MHBs (**Figure 2C**), though the difference was not significant (**Table 3**). The AUC for ALT > 1.5 × ULN at 12 weeks was 0.637, and its predictive value for HBsAg clearance was lower than that of LHBs and MHBs at 12 weeks.

**Table 3:** ROC curves for the prediction of HBsAg loss in patients.

Parameter	ROC curves					Comparison of AUC for indicators			
	AUC	95% CI	cut-off	sensitivity	specificity	indicators	difference between AUC	p value	95% CI
Baseline									
Total HBsAg (a)	0.654	0.521 - 0.773	114.7	61.54	66.67	–	–	–	–
LHBs (b)	0.827	0.707 - 0.912	13.99	87.18	61.90	a vs.b	0.172	0.072	-0.016 - 0.360
MHBs (c)	0.887	0.779 - 0.954	7.95	100.00	71.43	a vs.c	0.233	0.014	0.048 - 0.417
Combination factor 1(d)	0.922	0.823 - 0.975	-0.83	100.00	85.71	a vs.d	0.267	0.004	0.087 - 0.448
After 12 weeks of treatment									
Total HBsAg (e)	0.755	0.626 - 0.857	29.57	69.23	71.43	–	–	–	–
LHBs (f)	0.896	0.790 - 0.960	11.44	84.62	85.71	e vs.f	0.142	0.082	-0.018 - 0.301
MHBs (g)	0.863	0.750 - 0.938	5.84	64.10	100.00	e vs.g	0.109	0.179	-0.050 - 0.267
Combination factor 2(h)	0.939	0.846 - 0.984	1.00	82.05	100.00	e vs.h	0.184	0.015	0.037 - 0.332
Change from baseline at 12 weeks									
The reduction in total HBsAg (i)	0.691	0.559 - 0.804	0.29	74.36	61.90	–	–	–	–
The reduction in LHBs (j)	0.711	0.580 - 0.821	0.07	69.23	76.19	i vs.j	0.020	0.837	-0.172 - 0.212
The reduction in MHBs (k)	0.507	0.374 - 0.638	0.17	84.62	4.76	i vs.k	0.184	0.091	-0.029 - 0.398
ALT>1.5×ULN(l)	0.637	0.503 - 0.758	0.00	51.28	76.19	i vs.l	0.054	0.572	-0.133 - 0.240

HBsAg, Hepatitis B surface antigen; LHBs, large hepatitis B surface proteins; MHBs, middle hepatitis B surface proteins; ALT, alanine aminotransferase; ULN, upper limit of normal; AUC, area under the curve; CI, confidence interval.

To further improve the prediction accuracy, we used baseline LHBs (X1) and MHBs (X2) and 12-week LHBs (X3) and MHBs (X4) to construct joint prediction indicators. The logistic regression models and predictive probability equation (PRE) were as follows:


combination factor 1=−23.5594 + 0.84516 * X1 + 1.55683 * X2

$$\text{combination factor }1\text{ PRE}=\frac{1}{1+e^{-\left(-23.5594\;+\;0.84516\;*\text{ X}1\;+\;1.55683\;*\text{ X}2\right)}}$$

combination factor 2=−19.2726 + 0.81440 * X3 + 1.44604 * X4

$$\text{combination factor }2\text{ PRE}=\frac{1}{1+e^{-\left(-19.2726\;+\;0.81440\;*\text{ X}3\;+\;1.44604\;*\text{ X}4\right)}}$$

The results indicated that the AUCs for combination factor 1 and combination factor 2 constructed from LHBs and MHBs were significantly increased; the AUCs were 0.922 and 0.939, respectively (**Figures 2A, B**; **Table 3**). The predictive values of combination factor 1 and combination factor 2 for HBsAg clearance were comparable. The values of combination factor 1 and combination factor 2 in predicting HBsAg clearance were both higher than that of the total HBsAg of the corresponding time point, and the differences were statistically significant (p< 0.05, **Table 3**).

#### The predictive value of single and combined indicators for HBsAg clearance

**Figure 3:**
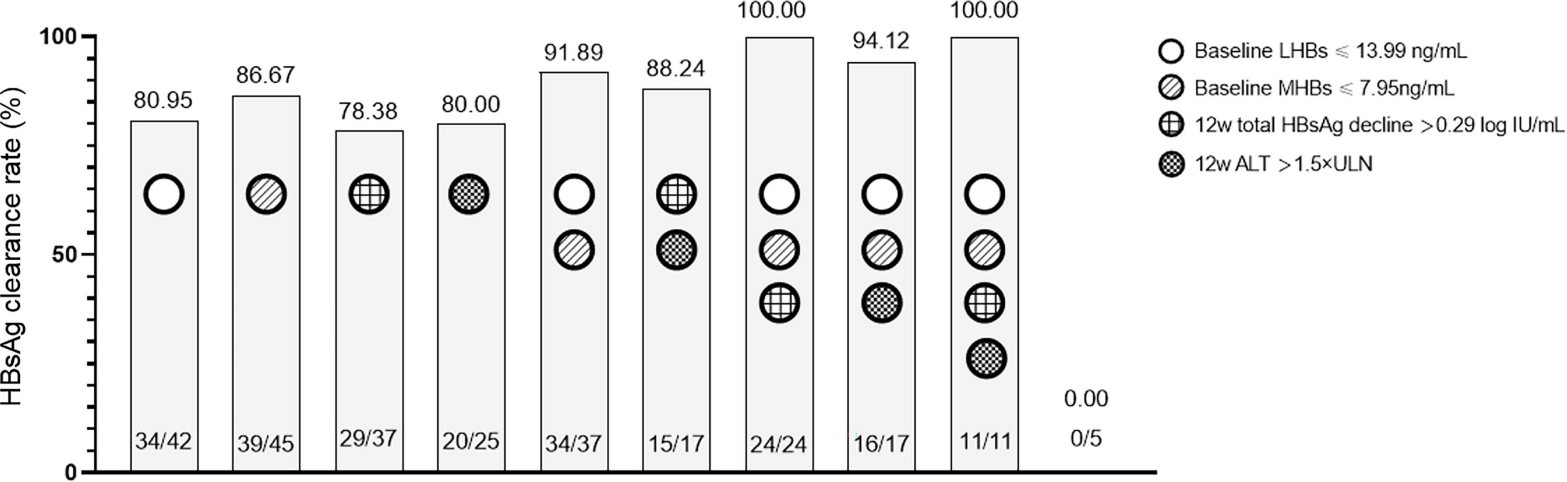
The predictive value of single and combined indicators for HBsAg clearance.

To more intuitively and conveniently apply the predictive indicators in clinical practice, we compared the difference between single indicator and combined indicator prediction. The analysis was performed based on the cut-off value for each indicator in **Table 3**. As shown in **Figure 3**, when a single indicator was used, the predictive values of baseline LHBs ≤ 13.99 ng/mL and MHBs ≤ 7.95 ng/ml for HBsAg clearance were good (>80.00%). When the combined indicators were used for prediction, the accuracy of the combined prediction of HBsAg clearance based on baseline LHBs ≤ 13.99 ng/mL and MHBs ≤ 7.95 ng/ml was higher than 90.00%. The combination of baseline LHBs ≤ 13.99 ng/mL, MHBs ≤ 7.95 ng/ml, and 12-week total HBsAg reduction > 0.29 log10 IU/ml predicted that the HBsAg clearance rate could reach 100%.

## Discussion

HBsAg clearance is currently the highest goal of clinical treatment for chronic hepatitis B, but it is still difficult to achieve using existing treatment regimens (2, 5). Screening the dominant population to administer precise treatment has important guiding significance in clinical practice. In the past, the total HBsAg level was often used to predict clinical cure. Some recent retrospective studies have found that LHB and MHB levels are correlated with HBV DNA response and HBeAg conversion (6–8). However, few studies have reported their correlation with HBsAg clearance. Therefore, this study analyzed the correlation between LHB and MHB levels and HBsAg clearance in an IHCs population treated with Peg-IFN.

The outer membrane protein of hepatitis B virus, namely, total HBsAg, is composed of three components: LHBs, MHBs, and small surface proteins. The 226 amino acid-long S domain comprises the SHBs and forms the carboxy-terminus in LHBs and MHBs. The preS2 domain is present in LHBs and MHBs, while the preS1 domain is present only in LHBs (6). The composition ratio of LHB and MHB levels at different stages of HBV infection is different. Pfefferkorn et al. reported that the baseline LHB and MHB levels in HBeAg-negative CHB were 2.5 ± 0.6 log10 ng/ml and 2.1 ± 0.8 log10 ng/ml, respectively; in HBeAg-positive CHB, the levels were 3.1 ± 0.6 log 10 ng/ml and 2.6 ± 0.8 log10 ng/ml, respectively (9). In this study, the levels of LHBs and MHBs at baseline in the 60 IHCs were significantly lower than those reported above, i.e., 13.28 ± 1.89 ng/ml (1.12 ± 0.06 log10 ng/ml) and 7.19 ± 1.19 ng/ml (0.85 ± 0.07 log10 ng/ml), respectively. This is similar to the reported by Peiffer KH and Liu C, who suggested that the proportion of LHBs and MHBs in IHCs was lower than that in HBeAg-negative or HBeAg-positive CHB patients (8, 10).

In this study, there were 39 patients in the R group and 21 patients in the NR group. The total HBsAg, LHB, and MHB levels at baseline were compared between the two groups. The results showed that the levels of total HBsAg, LHB, and MHB in the R group were significantly lower than those in the NR group (all p< 0.05, **Table 1**). The total HBsAg, LHB, and MHB levels in the two groups significantly decreased after Peg-IFN treatment, but the total HBsAg and LHB levels in the R group decreased more at 12 weeks of treatment (p = 0.021, **Figure 1B**; p = 0.015, **Figure 1D**). Recently, researchers studied HBeAg-positive CHB patients treated with Peg-IFN and reported a similar situation. Rinker et al. found that patients with low levels of LHBs and MHBs were more likely to have high HBeAg conversion after 24 weeks of treatment (6). Zhu et al. reported that patients with low LHB levels at 4 weeks were more likely to obtain HBeAg conversion (7). However, it is not clear whether LHBs and MHBs are associated with HBsAg clearance after Peg-IFN treatment.

It is generally believed that patients with good responsiveness to Peg-IFN are often accompanied by elevated ALT. Similar phenomena were also observed in this study. In patients with HBsAg clearance, the degree of ALT elevation was greater after 12 weeks of Peg-IFN treatment, and when ALT > 1.5 × ULN, the difference between the two groups was statistically significant (p = 0.04, **Table 1**). This reflects the double-edged sword effect of Peg-IFN treatment in both immune clearance and injury.

Regarding the predictive value of total HBsAg, LHB, and MHB levels for HBsAg clearance, the multivariate logistic regression analysis results indicated that baseline and 12-week LHB and MHB levels were independent predictors. Further ROC curve analyses were performed (**Figure 2**). The prediction results for a single indicator showed that the value of the baseline or 12-week LHB and MHB levels in predicting HBsAg clearance was greater than that of total HBsAg level at the corresponding time point, the 12-week reduction in total HBsAg or ALT >1.5 × ULN. To improve the prediction accuracy, a combined predictive factor was constructed through a logistic regression model (11, 12). Baseline LHBs and MHBs were combined to construct combination factor 1, and the 12-week LHBs and MHBs were combined to construct combination factor 2; the AUCs were 0.922 and 0.939, respectively, indicating that the value of predicting HBsAg clearance was significantly higher than the above single indicators. In view of the complex calculation of the combination factors, for the convenience of clinical use, we performed combined indicators analysis using the cut-off value for each predictive indicator (**Table 3**, **Figure 3**). The results showed that the combined indicators based on LHBs and MHBs predicted HBsAg clearance rates of more than 90.0%, among which, the combination of baseline LHBs ≤ 13.99 ng/ml, baseline MHBs ≤ 7.95 ng/ml, and 12-week total HBsAg reduction > 0.29 log10 IU/ml predicted HBsAg clearance rates can reach 100%. In this study, the commonly used clinical efficacy predictors of 12-week total HBsAg reduction (>0.29 log10 IU/ml) and ALT elevation (>1.5 × ULN) combined predicted an HBsAg clearance rate of 88.24%. Similar to our previous study, in IHCs treated with Peg-IFN, when HBsAg decreased > 0.30 log10 IU/ml and ALT elevation (greater than ULN) at 12 weeks were combined, the predicted HBsAg clearance rate was 80.80% (13). The traditional indicators can predict HBsAg clearance, but their predictive value is lower than that of LHBs and MHBs. As seen in **Table 3**, the combined indicators have higher sensitivity and specificity in predicting HBsAg clearance. But the prediction values (sensitivity and specificity) were without validation from external data, the models could be over fitted based on the sample size. Further studies are need in the future to confirm the prediction role of Large and Medium Surface Proteins.

LHBs are necessary for the entry of HBV into hepatocytes and the secretion of viral particles from hepatocytes (14, 15). MHBs play a regulatory role in HBV replication, and MHB deletion can affect viral particle secretion (16). In 1987, Gerken et al. (17) demonstrated that a rapid decline in LHBs and MHBs occurred during the early stage of spontaneous HBsAg clearance in patients with acute hepatitis B. At 8 weeks after onset, 90% (18/20) of the patients were negative for serum MHBs, and at 16 weeks, 90% (18/20) of the patients had undetectable levels of LHBs. A similar phenomenon was also observed in the treatment of patients with chronic hepatitis B. Pfefferkorn et al. provided antiviral treatment to 83 patients with CHB, of whom 20 achieved HBsAg clearance (3 patients were in the Peg-IFN group and 17 patients were in the NA group). The results of that study suggested that the proportion of LHBs and MHBs in total HBsAg at baseline was significantly lower in HBsAg-cleared patients than in non-cleared patients and that the decrease in LHBs and MHBs was faster during treatment in HBsAg-cleared patients. The value of the proportion of baseline MHBs in predicting HBsAg clearance (AUC: 0.726) was higher than that of total HBsAg (AUC: 0.390) (4). The results were similar to those of our study. Not only did baseline and 12-week LHBs and MHBs have higher prediction value than total HBsAg in single indicator prediction, but the combination of LHBs and MHBs had better prediction value for HBsAg clearance. Compared with Pfefferkorn et al.’s study, in this study, LHBs and MHBs were better for predicting HBsAg clearance. The possible reason is that the patients included in this study were all IHCs patients, while Pfefferkorn et al. enrolled HBeAg-positive patients. In addition, the 39 patients with HBsAg clearance in our study were all underwent Peg-IFN treatment, while most of the patients with HBsAg clearance in the study by Pfefferkorn et al. underwent NA treatment. In addition, Rinker et al. studied 127 HBeAg-positive patients who received Peg-IFN treatment and found that LHBs and MHBs were similar to total HBsAg in predicting HBeAg conversion and did not have greater advantages (6).

In summary, the predictive value of LHB and MHB levels for HBsAg clearance was good and was better than total HBsAg level at both baseline and 12 weeks. In addition, the combination of LHBs and MHBs had a higher predictive value than did single factors, and the accuracy of predicting HBsAg clearance based on the combined baseline LHBs and MHBs was over 90.00%. Therefore, based on the current treatment strategy, the monitoring of LHBs and MHBs in the process of pursuing HBsAg clearance can provide a reference for clinical treatment. In addition, the detection of LHBs and MHBs is convenient and reproducible, which is important for clinical application. However, this study enrolled an IHCs population treated with Peg-IFN; therefore, the significance of the results needs to be further explored in a wider range of CHB patients.

## Data availability statement

The raw data supporting the conclusions of this article will be made available by the authors, without undue reservation.

## Ethics statement

The study was approved by the ethics committee of Beijing Youan Hospital affiliated with Capital Medical University ([2017]24). The patients/participants provided their written informed consent to participate in this study.

## Author contributions

XL, YZ, HL, ZC, and XC designed the study. JL, SR, YL, and XW collected the data. XL, YZ, and HL analyzed the data. SZ and LM guided statistical analysis. XL, YZ, and HL drafted the manuscript. ZC and XC contributed to the interpretation of the results and critical revision of the manuscript for important intellectual content. All authors contributed to the article and approved the submitted version.

## Funding

This work was supported by the Capital Clinical Diagnostic Techniques and the Translational Application Projects (Z211100002921059), Chinese National  Natural Science Foundation (81900537), Beijing Hospitals Authority Clinical medicine Development of special funding support (XMLX202125), and the Capital Health Research and Development Projects (2020–1–2181).

## Conflict of interest

The authors declare that the research was conducted in the absence of any commercial or financial relationships that could be construed as a potential conflict of interest.

The handling editor WY declared a shared parent affiliation with the authors during the review.

## Publisher’s note

All claims expressed in this article are solely those of the authors and do not necessarily represent those of their affiliated organizations, or those of the publisher, the editors and the reviewers. Any product that may be evaluated in this article, or claim that may be made by its manufacturer, is not guaranteed or endorsed by the publisher.

## Abbreviations

HBV, hepatitis B virus; CHB, chronic Hepatitis B; HBsAg, hepatitis B surface antigen; LHBs, large hepatitis B surface proteins; MHBs, middle hepatitis B surface proteins; NAs, nucleos(t)ide analogue; Peg-IFN, pegylated interferon; IHCs, inactive HBsAg carriers; HBeAg, hepatitis B e antigen; ALT, alanine aminotransferase; ULN, upper limit of normal; ROC, receiver operating characteristic; OR, odds ratio; AUC, area under the curve; CI, confidence interval.
